# Non-invasive venous waveform analysis and the IntraVAscular volume Number (IVAN) track acute hemorrhage and volume overload in a piglet model

**DOI:** 10.21203/rs.3.rs-10501784/v1

**Published:** 2026-08-13

**Authors:** Jenna H. Sobey, Srijaya K. Reddy, Asif Padiyath, Bryce Perrien, Christina Jelly, Lexie H. Vaughn, Monica Polcz, Dawson Wervey, Romy Pein, Danny Jeong, Tara Isanaka, Amy L. Friedman, Anna B. Galloway, Angela J. Weingarten, Joyce Cheung-Flynn, Colleen Brophy, Eric Wise, Marisa Case, Kyle M. Hocking, Bret D. Alvis

**Affiliations:** Monroe Carell Jr. Children’s Hospital at Vanderbilt; Monroe Carell Jr. Children’s Hospital at Vanderbilt; University of Pennsylvania, Children’s Hospital of Philadelphia; Vanderbilt University Medical Center; Vanderbilt University Medical Center; University of Arkansas for Medical Sciences; University of South Florida; Vanderbilt University Medical Center; Vanderbilt University Medical Center; Vanderbilt University Medical Center; Vanderbilt University Medical Center; Vanderbilt University Medical Center; Monroe Carell Jr. Children’s Hospital at Vanderbilt; Monroe Carell Jr. Children’s Hospital at Vanderbilt; Vanderbilt University Medical Center; Vanderbilt University Medical Center; University of Minnesota; Vanderbilt University Medical Center; Vanderbilt University Medical Center; Vanderbilt University Medical Center

**Keywords:** blood volume, child, fluid therapy, hemorrhage, monitoring, physiological, venous waveform

## Abstract

**Purpose.:**

Acute intravascular volume change is difficult to detect in children; most hemodynamic monitors were developed in adults. Non-invasive venous waveform analysis (NIVA) derives the IntraVAscular volume Number (IVAN) from the peripheral venous waveform. We evaluated whether NIVA tracks acute volume change across hypovolemia and hypervolemia in a pediatric-scale porcine model.

**Methods.:**

Eight anesthetized, mechanically ventilated piglets underwent controlled hemorrhage (to 5%, 10%, 15%, 20%, and 25% of estimated blood volume, or until a 20% reduction in mean arterial pressure), then reinfusion and crystalloid-induced volume overload. NIVA was acquired simultaneously with invasive measurements, and IVAN was compared with volume change and invasive measures using correlation and concordance analyzes.

**Results.:**

Data were analyzable for seven piglets (hemorrhage) and four piglets (overload). Across the combined range, IVAN tracked volume change with 92% concordance (r = 0.68; P < 0.0001), comparable to right atrial pressure (98%, r = 0.69; P < 0.0001) and pulmonary capillary wedge pressure (89%, r = 0.65; P < 0.0001) and markedly superior to mean arterial pressure (67%, r = − 0.12; P = 0.32). Raw IVAN discriminated 15% and 25% blood loss (area under the ROC curve 0.87 and 0.89; paired reductions P = 0.016 and P = 0.047). Glucose, lactate, and creatinine also tracked volume change; most blood-gas and electrolyte indices did not.

**Conclusion.:**

In this pediatric-scale porcine model, IVAN tracked intravascular volume change comparably to invasively measured filling pressures and substantially better than mean arterial pressure, supporting further development for pediatric volume monitoring.

## Introduction

Accurate assessment of intravascular volume status is a central and unmet challenge in the perioperative and critical care of infants and children.[1, 2] During surgical hemorrhage, pediatric patients maintain their conventional vital signs until a large fraction of blood volume has been lost, such that overt hemodynamic changes often appear only after significant blood loss and end-organ injury have already occurred.[3] The Pediatric Perioperative Cardiac Arrest (POCA) Registry identifies under-resuscitation of hemorrhage as one of the principal causes of perioperative death in children.[4, 5] The opposite error is equally consequential: excessive fluid administration and resulting volume overload are independently associated with increased morbidity and mortality in critically ill children.[6, 7] Clinicians are therefore asked to keep patients within a narrow therapeutic window, yet the available tools are poorly suited to the task. Clinical examination and routine monitoring are insensitive to acute volume change,[1, 3] invasive measures such as central venous or pulmonary capillary wedge pressure are impractical and carry risk in small patients,[5] and echocardiographic surrogates are frequently inaccessible in the operative field and require an adequately trained/skilled provider.[1, 2]

The majority of monitoring devices and volume-assessment algorithms were designed, trained, and validated in adults and then applied to children with little adaptation, despite fundamental physiological differences between the populations.[2, 8] Infants and young children exhibit greater arterial compliance and a wide range of ages, sizes, and weights, so metrics derived from adult physiology cannot be assumed to correlate in children.[1, 2] Pediatric device development lags markedly behind that of adults; only a minority of devices cleared by the U.S. Food and Drug Administration carry a pediatric indication, and most of those are limited to older children.[9] There is consequently a clear need not merely to borrow adult tools for pediatric use, but to develop and optimize monitoring technologies specifically for the pediatric population, using pediatric-relevant physiological reference standards from the outset.[9]

Non-invasive venous waveform analysis (NIVA) is an emerging approach that addresses this need directly.[8, 10] Rather than relying on absolute venous pressure, NIVA uses a piezoelectric sensor to non-invasively capture the peripheral venous waveform, which is then decomposed into its time- and frequency-domain features for analysis and quantification of intravascular volume.[8, 10] From these features, a summary index, the IntraVAscular volume Number (IVAN), is derived to track changes in volume.[10, 11] In adults, NIVA-derived measures correlate with pulmonary capillary wedge pressure and are sensitive to small reductions in blood volume in healthy blood donors,[10, 11, 12] and preliminary pediatric data, including patients undergoing complex cranial vault reconstruction and pediatric heart transplant recipients studied in the catheterization laboratory, suggest that NIVA harmonics correlate with acute volume changes and invasively measured filling pressures, respectively.[13, 14] To truly understand and adapt venous waveform analysis to the pediatric patient, NIVA and IVAN for pediatric use requires development and validation against controlled, measurable changes in intravascular volume within a pediatric-scale physiological model.

A controlled large-animal model provides the volume manipulation and reference measurements needed for this continued development. The piglet approximates the size and cardiovascular physiology of a young child while permitting precise, standardized changes in intravascular volume that cannot be ethically produced in pediatric subjects. In this study, we applied a standardized porcine model of acute hemorrhage and volume overload in eight piglets, acquiring NIVA signals continuously and simultaneously with invasive hemodynamic measurements across graded blood withdrawal, resuscitation, and crystalloid-induced volume overload. We hypothesized that IVAN would track acute changes in intravascular volume across both hypovolemic and hypervolemic states in a pediatric-scale model similar to the results witnessed in children undergoing complex cranial vault reconstruction.[13]

## Methods

### Ethical approval and animals

This study was approved by the Vanderbilt University Institutional Animal Care and Use Committee, and all procedures were performed in accordance with institutional and national guidelines for the care and use of laboratory animals. Reporting follows the ARRIVE 2.0 guidelines. Eight Yorkshire piglets of both sexes, approximately 5 weeks of age (developmentally comparable to a human infant) and weighing 4–10 kg (mean approximately 7 kg), obtained from a commercial vendor, were studied.

### Anesthesia and ventilation

Anesthesia was induced through an auricular (ear) vein catheter with ketamine (2.2 mg kg^−1^), xylazine (2.2 mg kg^−1^), tiletamine–zolazepam (4.4 mg kg^−1^), and dexmedetomidine (0.005 mg kg^−1^). The trachea was intubated under direct laryngoscopy with a 4.5–5.5 mm cuffed endotracheal tube, and anesthesia was maintained with 1% isoflurane, supplemented as required with intravenous dexmedetomidine (0.005–0.01 mg kg^−1^ every 2 h). Animals received volume-controlled ventilation with a tidal volume of 8 ml kg^−1^, respiratory rate titrated to an end-tidal carbon dioxide of 35–40 mmHg, and positive endexpiratory pressure of 5 cmH_2_O; the tidal volume of 8 ml kg^−1^ standardized conditions for measurement of pulse pressure variation.

### Instrumentation and monitoring

An arterial catheter was placed in the carotid artery for continuous measurement of mean and systolic arterial pressure and pulse pressure variation. A central catheter was placed in the internal jugular vein (Fig. 1) and a pulmonary artery catheter (PAC; Fig. 1) was placed through the central catheter’s introducer. Piezoelectric NIVA sensors were positioned on the lateral aspect of an upper extremity (Fig. 1A). Hemodynamic signals were recorded with LabChart software on a PowerLab system (ADInstruments, Sydney, Australia) with synchronised time stamps to permit accurate alignment of NIVA and reference data. Transthoracic echocardiography was performed (Fig. 1C; Butterfly Network, Inc., Burlington, MA, USA) before and after each change in blood volume to record the aortic peak systolic velocity at end-inspiration and end-expiration, from which the respiratory variation in peak velocity (ΔVpeak) was calculated as 100 × (maximum − minimum)/mean.

### Controlled hemorrhage

After systemic heparinisation, controlled hemorrhage was produced by withdrawing blood with a 30 ml syringe (Fig. 1B). Estimated blood volume was calculated as 80 ml kg^−1^. Hemodynamic measurements and NIVA signals were obtained at 5%, 10%, 15%, 20%, and 25% of estimated blood volume removed, or until a 20% decrease in mean arterial pressure (MAP), whichever occurred first.

### Resuscitation and volume overload

Animals were resuscitated by reinfusion of the shed blood at 20 ml min^−1^ and allowed to equilibrate for 30 min before repeat measurements. A state of volume overload was then produced by administering balanced crystalloid (Plasma-Lyte; Baxter Healthcare Corporation, Deerfield, IL, USA) in 20 ml kg^−1^ boluses over 10 min until 500 ml had been administered or cardiac output decreased by 15%. Vital signs, hemodynamic parameters, haematocrit, and urine output were recorded concurrently at each volume state.

### NIVA signal processing and signal-quality criteria

Raw peripheral venous waveforms were acquired from the piezoelectric sensor and recorded in LabChart software at a sampling rate of 500 Hz. Signals were converted from the time domain to the frequency domain using a fast Fourier transform with a 16,384-sample (16k) window, and the peak powers of the fundamental frequency (f_0_) and its harmonics (f_0_–f_7_) were extracted. A proprietary ratiometric algorithm combined the relative power contributions of these frequencies to derive the IntraVAscular volume Number (IVAN), with weighting optimized by least-squares analysis against the measured change in volume.[12] A pre-specified signal-quality threshold was applied before analysis: recording segments containing large-amplitude artifactual spikes were excluded, and a segment was required to reach analysis completion within 90,000 samples (approximately 180 s at 500 Hz) to be retained. Segments not meeting these criteria were excluded from the corresponding phase of analysis.

### Statistical analysis

The primary analysis evaluated directional concordance between change in each hemodynamic measure (IVAN, right atrial pressure [RAP], pulmonary capillary wedge pressure [PCWP], and MAP) and change in intravascular volume, expressed as the percentage of estimated blood volume removed or added relative to baseline. Concordance was defined as the proportion of paired observations in which the measure and the intravascular volume changed in the same direction, and the strength of association was quantified with the Pearson correlation coefficient. The ability of raw IVAN and RAP to discriminate defined volume states (15% and 25% blood loss) from baseline was assessed by receiver-operating-characteristic (ROC) analysis (one value per animal), and paired baseline-to-event changes were tested with the Wilcoxon matched-pairs signed-rank test. Secondary analyzes correlated the intravascular volume change with concurrently measured laboratory, arterial and mixed-venous blood-gas, and echocardiographic variables using Pearson correlation; unlike the NIVA concordance analysis, these secondary analyzes were not restricted to the animals with complete analyzable NIVA data and the number of piglets contributing to each correlation, which varied by variable and phase, is reported in Table 1. Analyses were performed for the hemorrhage phase, the volume-overload phase, and the combined data set. A two-tailed P < 0.05 was considered significant. Analyses were performed in GraphPad Prism (GraphPad Software, Boston, MA, USA); paired observations were pooled across animals for the correlation and concordance analyzes. A formal power calculation is not well established for repeated-measures correlation designs of this type; on the basis of a prior controlled porcine study of venous waveform-based volume monitoring[15] and the repeated-measures structure, in which each animal contributes multiple paired observations across graded volume states, a minimum of five evaluable piglets was estimated to provide adequate paired observations to detect a moderate association (r ≈ 0.5) between IVAN and intravascular volume change. To allow for the data-capture failure and signal-quality exclusions expected with a prototype device, nine piglets were enrolled, of which eight completed the standardized protocol.

## Results

### Animals and analyzable data

Eight piglets underwent the standardized protocol. After application of the pre-specified signal-quality criteria, complete analyzable NIVA data were available for seven piglets during the hemorrhage phase and for four piglets during the volume-overload phase. Four piglets contributed hemorrhage data only, because the device either failed to record the overload phase or the recording did not meet the analysis-quality threshold; three piglets contributed both phases; and one piglet contributed overload data only, because the hemorrhage recording did not meet the quality threshold. Hemorrhage data were censored beyond 25% of estimated blood volume removed, as fewer than half of the analyzed piglets had retained data points beyond this level.

### IVAN tracks directional volume change and performs comparably to invasively measured filling pressures

Across the combined hemorrhage and volume-overload range (25% of estimated blood volume removed to 100% relative volume expansion), change in IVAN moved in the same direction as change in intravascular volume in 49 of 53 paired observations, a directional concordance of 92% (Fig. 2; Pearson r = 0.68, P < 0.0001). This performance was comparable to that of the invasively measured filling pressures measured simultaneously: change in RAP showed 98% concordance (Fig. 3B; r = 0.69, P < 0.0001) and change in PCWP showed 89% concordance (Fig. 3C; r = 0.65, P < 0.0001). In marked contrast, change in MAP, the non-invasive measure most often relied upon clinically as a surrogate for volume status, showed only 67% concordance and did not correlate with intravascular volume change (Fig. 3A; r = − 0.12, P = 0.32). Considered by phase, IVAN concordance with volume change was 92% during hemorrhage (36 of 39 paired observations; r = 0.38) and 93% during volume overload (13 of 14 paired observations; r = 0.65).

### IVAN discriminates graded blood loss from baseline

Raw IVAN values (one value per animal, n = 7) discriminated defined degrees of blood loss from baseline. The area under the ROC curve was 0.87 for 15% blood loss and 0.89 for 25% blood loss. Paired reductions in IVAN from baseline to each volume state were significant (Wilcoxon matched-pairs signed-rank test: P = 0.016 at 15% loss and P = 0.047 at 25% loss). Invasive RAP performed similarly, with an area under the ROC curve of 0.88 at both 15% and 25% blood loss and significant paired reductions (P = 0.016 at both states).

### Physiological, laboratory, and echocardiographic correlates of volume change

Because this model captured serial invasive pressure measurements, arterial and pulmonary-arterial blood gases, laboratory chemistry, and echocardiographic indices at each volume state, we examined which of these tracked the intravascular volume change (Table 1). The respiratory variation in aortic peak systolic velocity (ΔVpeak), a validated dynamic index of fluid responsiveness,[1, 16] did not track intravascular volume change in any phase (hemorrhage r = − 0.07; overload r = 0.12; combined r = − 0.01; all P > 0.4). During the hemorrhage phase, no laboratory, blood-gas, or echocardiographic parameter significantly tracked volume change; lactate approached significance (r = − 0.33, P = 0.051) and glucose and creatinine showed weak, non-significant negative trends (r = − 0.22, P = 0.21 and r = − 0.27, P = 0.12, respectively), whereas hemoglobin, electrolytes, pH, and blood-gas tensions showed no association with this acute volume loss (all P > 0.3). During volume overload, glucose (r = − 0.61, P = 0.006), potassium (r = − 0.46, P = 0.045), and creatinine (r = − 0.47, P = 0.044) tracked volume change, and hemoglobin concentration fell as volume was added, approaching significance (r = − 0.43, P = 0.067) in keeping with haemodilution; in the combined data set glucose (r = − 0.51, P < 0.0001), creatinine (r = − 0.37, P = 0.005), lactate (r = − 0.30, P = 0.025), and oxygen saturation (r = − 0.29, P = 0.032) reached significance. Notably, most conventional blood-gas and electrolyte indices, including pH, arterial carbon dioxide and oxygen tensions, bicarbonate, base excess, sodium, and calcium, did not significantly track acute volume change in any phase, and pulmonary-arterial oxygen saturation approached but did not reach significance (r = 0.36, P = 0.058).

## Discussion

In a pediatric-scale porcine model of acute hemorrhage and iatrogenic volume overload, the non-invasive IVAN index tracked the direction of intravascular volume change with 92% concordance and correlated with invasively measured filling pressures. Changes in MAP lagged behind both the IVAN number and measured filling pressures. IVAN also discriminated 15% and 25% blood loss from baseline, with areas under the ROC curve of 0.87 and 0.89.

Invasively measured hemodynamics are the gold standard for assessment of volume status. RAP tracked volume change most closely (98% concordance, r = 0.69; area under the ROC curve 0.88), consistent with central venous filling falling with hemorrhage and rising with volume loading. PCWP, the classic gold standard for left-sided filling, also tracked volume well (89% concordance, r = 0.65). Together, they establish the achievable ceiling for directional volume tracking in this model and the reference against which a non-invasive surrogate should be judged.

Against that reference, IVAN correlated well with invasively measured filling pressures (92% concordance, r = 0.68 across the combined range, falling between PCWP and RAP). The contrast with MAP is equally important; MAP showed only 67% concordance and a slightly negative correlation (r = − 0.12), reflecting arterial pressure's insensitivity to volume until compensation is exhausted.[18] This dissociation, a venous-derived index tracking volume while arterial pressure does not, reproduces our earlier proof-of-concept observation in children undergoing complex cranial vault reconstruction.[13] The present study extends that observation into a controlled model with simultaneous invasive references and a defined bidirectional volume challenge, showing that the venous signal tracks volume comparably to the invasive standards themselves. One nuance deserves emphasis: the two limbs of the concordance plots are not physiologically equivalent. Whole-blood withdrawal removes intravascular volume directly, whereas crystalloid distributes across the extracellular space, so equal percentages of estimated blood volume produce unequal intravascular change. Directional concordance nonetheless remains valid and valuable, because the direction of volume change is unambiguous in both phases.

A distinctive strength of this work is the breadth of simultaneous reference data captured in a single small-animal preparation, spanning invasively measured pressures (RAP and PCWP), arterial and pulmonary-arterial blood gases, serial laboratory chemistry, and echocardiographic indices across both a controlled hemorrhage and a controlled volume-overload sequence in the same animals. Metabolic markers behaved as physiology predicts but imperfectly: glucose and creatinine tracked volume most clearly across the overload phase, whereas lactate tracked only in the combined data set and was borderline during hemorrhage. Hemoglobin declined as volume was loaded, confirming genuine intravascular expansion. Notably, ΔVpeak, a dynamic echocardiographic index validated for fluid responsiveness in ventilated children,[1] did not track volume, because it was designed to predict whether a patient will respond to a fluid bolus, indexing the position on the Frank-Starling curve through heart-lung interaction, rather than to quantify the direction or magnitude of a volume change; its performance also depends on tidal volume, compliance, heart rate, and body size. IVAN, by contrast, tracks the volume change itself, so the two measure related but distinct quantities. Just as instructive were the negative findings: most blood-gas and electrolyte indices, including pH, carbon dioxide and oxygen tensions, bicarbonate, base excess, sodium, and calcium, did not significantly track acute volume change, reinforcing the rationale for a dedicated volume monitor.

Several limitations temper these conclusions. The sample is small: seven piglets contributed hemorrhage data and only four contributed complete volume-overload data, so the overload findings are preliminary. This size is nonetheless consistent with published controlled porcine studies of volume monitoring, in which dense repeated within-animal measurements yield many paired observations from few animals, including a directly analogous eight-animal venous-waveform study of hemorrhage and volume overload.[15, 17] Significant correlations were still detected in the overload phase, indicating adequate paired observations. Data-capture failures and a pre-specified signal-quality threshold removed some recordings, reducing power and reflecting the maturity of a prototype device, and hemorrhage data were censored beyond 25% blood volume owing to hemodynamic instability. The animals also represented a single, narrow developmental stage (approximately 5 weeks of age, comparable to a human infant), and performance across the pediatric spectrum of size and age remains to be established. Finally, this single-species, anesthetized, ventilated model and the pooling of repeated observations without modeling within-animal clustering may overstate precision; directional concordance does not establish the limits of agreement a bedside monitor will ultimately require, and the secondary correlations are exploratory and unadjusted for multiple comparisons.

In conclusion, in this pediatric-scale porcine model of acute hemorrhage and volume overload, IVAN tracked directional volume change comparably to invasive right atrial and pulmonary capillary wedge pressures and substantially better than mean arterial pressure. With prior human proof-of-concept data, these results support quantitative validation of non-invasive venous waveform analysis as a pediatric volume monitor and motivate a larger study powered to establish agreement and reliable data capture.

## Figures and Tables

**Figure 1:**
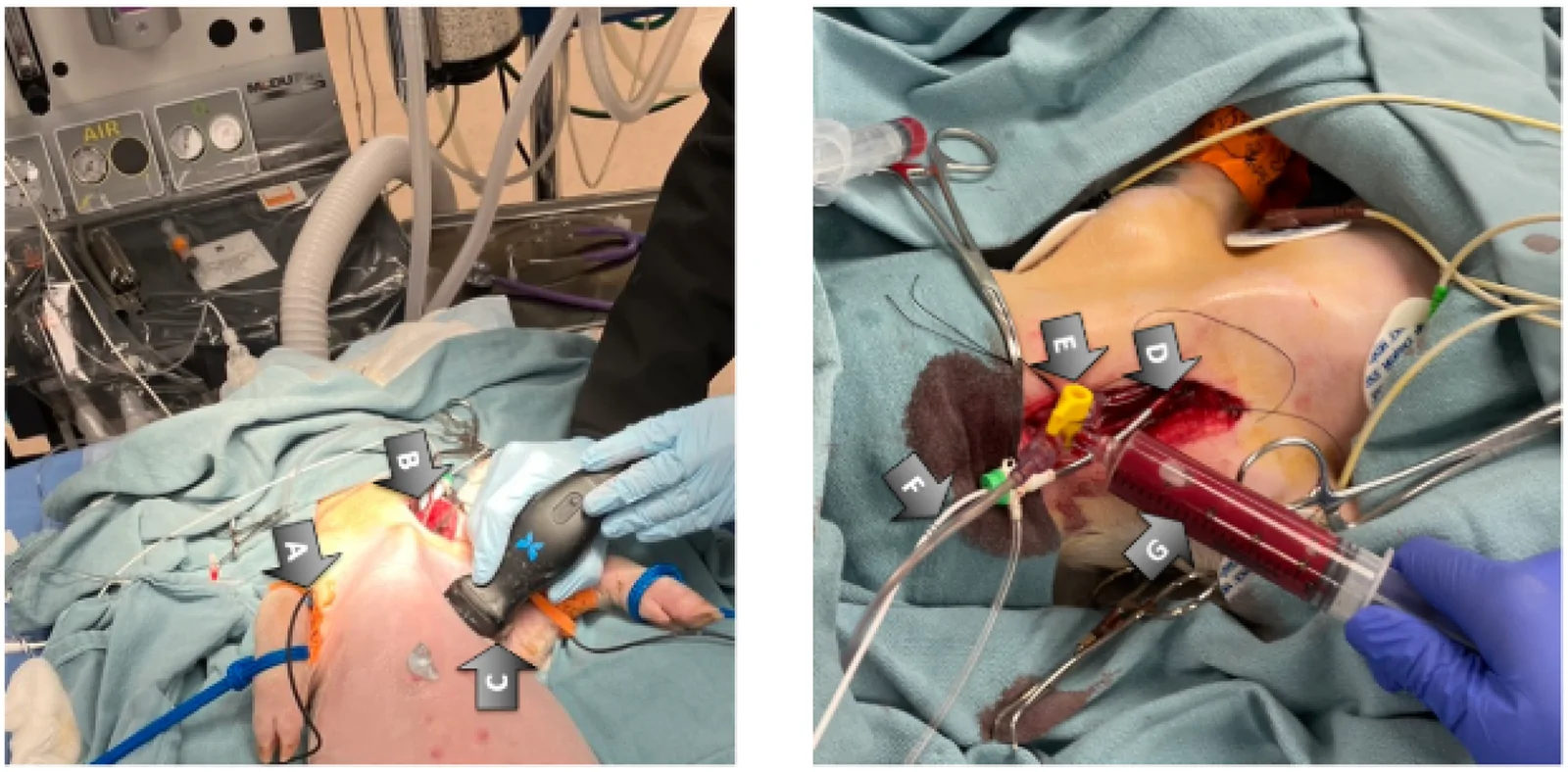
Non-invasive venous waveform analysis (NIVA) device and sensor placement for peripheral venous waveform acquisition along with invasive monitoring in piglets. Photograph of the NIVA data-acquisition setup (Left Picture), showing the piezoelectric sensor applied over a peripheral vein (A), adjacent vascular access (B), and transthoracic echocardiography probe (C). Close-up photograph of the invasive monitoring lines (Right Picture), showing the internal jugular access (D), the carotid arterial pressure monitor (E), and the pulmonary artery catheter (F). The syringe (G) was used to remove and administer volume. The sensor non-invasively captures the peripheral venous waveform, from which the IntraVAscular volume Number (IVAN) is derived. Abbreviations: IVAN, IntraVAscular volume Number; NIVA, non-invasive venous waveform analysis.

**Figure 2:**
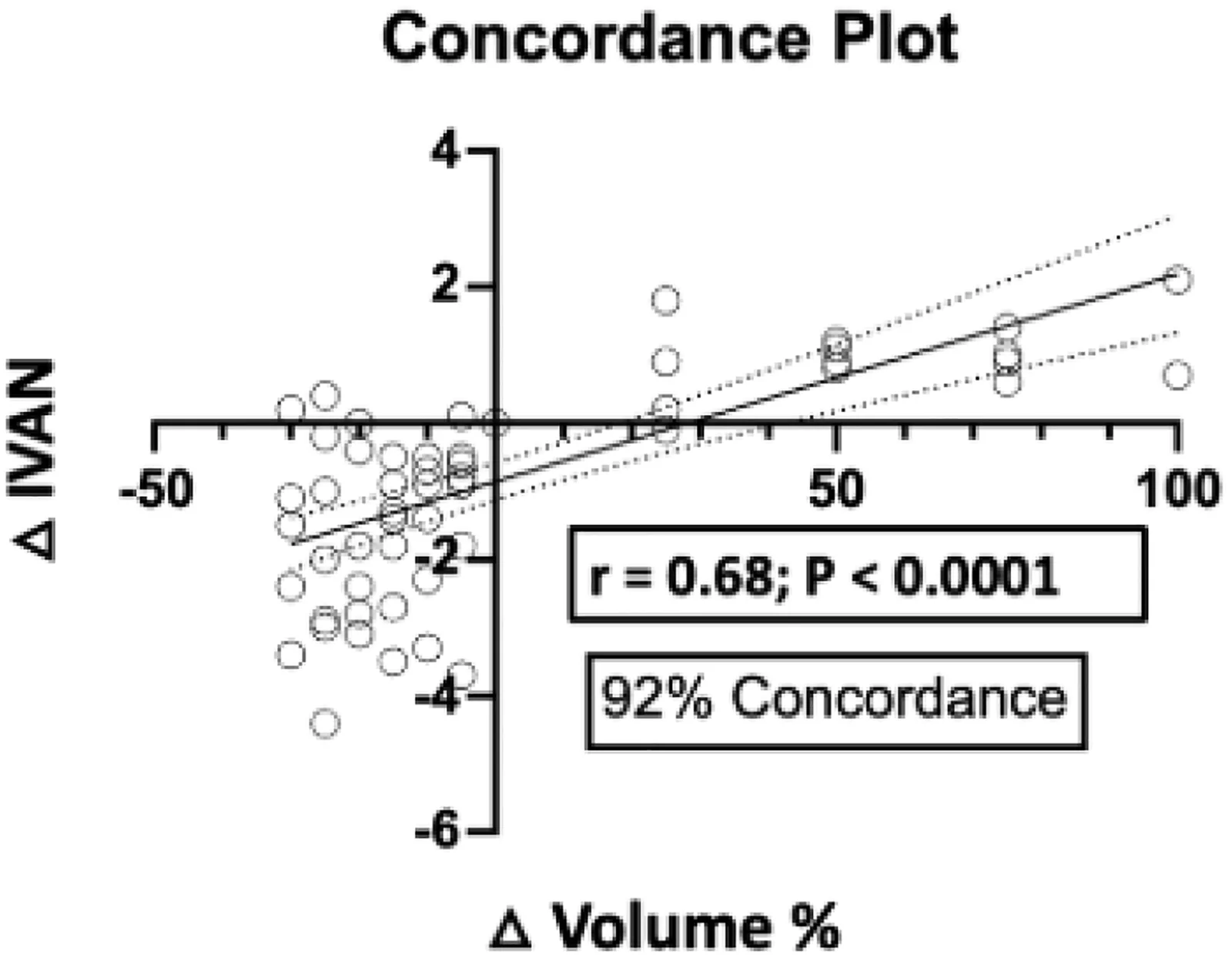
Concordance plot for the change in IVAN compared to change in intravascular volume. Concordance plot of the change in IVAN against the change in intravascular volume (percentage of estimated blood volume removed or added) across the combined hemorrhage and volume-overload range in piglets (n= 7 hemorrhage & 4 overload). Each point is a paired observation at a defined volume state, and the solid line is the linear regression with 95% confidence bands. Change in IVAN corresponded to the direction of intravascular volume change in 92% of paired observations (Pearson r = 0.68, P < 0.0001). Abbreviations: IVAN, IntraVAscular volume Number.

**Figure 3:**
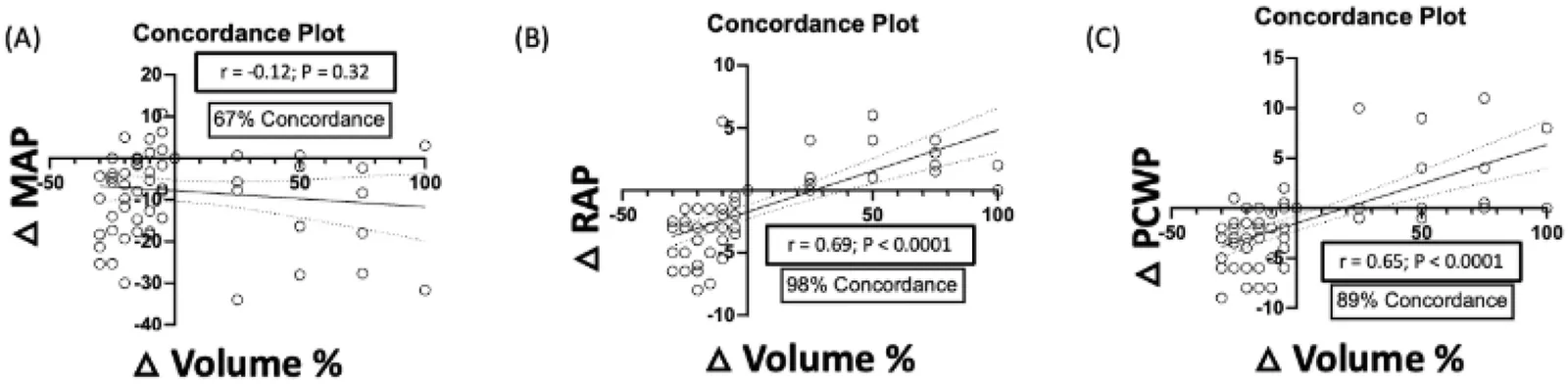
Mean arterial pressure and Invasive filling pressures compared to intravascular volume change. Concordance plots of the change in (A) mean arterial pressure (MAP), (B) right atrial pressure (RAP), and (C) pulmonary capillary wedge pressure (PCWP) against the change in intravascular volume (percentage of estimated blood volume removed or added) across the combined hemorrhage and volume-overload range in piglets (n= 7 hemorrhage & 4 overload). Change in RAP (B; 98% concordance, r = 0.69, P < 0.0001) and PCWP (C; 89% concordance, r = 0.65, P < 0.0001) tracked intravascular volume change, whereas MAP did not (A; 67% concordance, r = −0.12, P = 0.32). Each point is a paired observation, and the solid line is the linear regression with 95% confidence bands. Abbreviations: MAP, mean arterial pressure; PCWP, pulmonary capillary wedge pressure; RAP, right atrial pressure.

**Table 1:** Pearson correlations (r, two-tailed P) between the change in intravascular volume (% estimated blood volume removed or added) and the concurrent change (Δ) in each variable, by phase (hemorrhage, overload, combined).

VARIABLE	HAEMORRHAGE	OVERLOAD	COMBINED
Δ IVAN (NIVA)	**r = 0.38**	**r = 0.65**	**r = 0.68**
	(P = 0.017, n = 7)	(P = 0.012, n = 4)	(P < 0.0001, n = 7)
Aortic ΔVpeak (%)	**r = - 0.07**	**r = 0.12**	**r = - 0.01**
	(P = 0.60, n = 7)	(P = 0.48, n = 6)	(P = 0.94, n = 7)
Δ Glucose (mg dL^−1^)	**r = - 0.22**	**r = - 0.61**	**r = - 0.51**
	(P = 0.21, n = 5)	(P = 0.006, n = 4)	(P < 0.0001, n = 6)
Δ Creatinine (mg dL^−1^)	**r = - 0.27**	**r = - 0.47**	**r = - 0.37**
	(P = 0.12, n = 5)	(P = 0.044, n = 4)	(P = 0.005, n = 6)
Δ Lactate (mmol L^−1^)	**r = - 0.33**	**r = - 0.17**	**r = - 0.30**
	(P = 0.051, n = 5)	(P = 0.48, n = 4)	(P = 0.025, n = 6)
Δ Potassium (mmol L^−1^)	**r = 0.11**	**r = - 0.46**	**r = 0.22**
	(P = 0.54, n = 5)	(P = 0.045, n = 4)	(P = 0.12, n = 6)
Δ Hemoglobin (cHgb, g dL^−1^)	**r = 0.09**	**r = - 0.43**	**r = - 0.03**
	(P = 0.62, n = 5)	(P = 0.067, n = 4)	(P = 0.80, n = 6)
Δ Oxygen saturation (cSO2, %)	**r = - 0.17**	**r = - 0.39**	**r = - 0.29**
	(P = 0.35, n = 5)	(P = 0.099, n = 4)	(P = 0.032, n = 6)
Δ pH	**r = - 0.15**	**r = 0.05**	**r = - 0.06**
	(P = 0.39, n = 5)	(P = 0.84, n = 4)	(P = 0.67, n = 6)
Δ Arterial PaO2 (mmHg)	**r = 0.02**	**r = - 0.05**	**r = 0.14**
	(P = 0.90, n = 5)	(P = 0.83, n = 4)	(P = 0.31, n = 6)
Δ Arterial pCO2 (mmHg)	**r = 0.10**	**r = - 0.16**	**r = 0.04**
	(P = 0.58, n = 5)	(P = 0.50, n = 4)	(P = 0.76, n = 6)

n, number of piglets. Analyses are exploratory and unadjusted for multiple comparisons; full matrices are provided in the Supplementary material. Units are in parentheses in the first column. Δ, change from baseline; IVAN, IntraVAscular volume Number derived from non-invasive venous waveform analysis (NIVA); cHgb, calculated hemoglobin; cSO2, calculated oxygen saturation; PaO2, arterial partial pressure of oxygen; pCO2, partial pressure of carbon dioxide; ΔVpeak, respiratory variation in aortic peak systolic velocity.

## Data Availability

The datasets generated and analyzed during the current study are available from the corresponding author upon reasonable request.
